# Acute fatty liver of pregnancy causes severe acute pancreatitis and stillborn fetus: A case report

**DOI:** 10.1097/MD.0000000000025524

**Published:** 2021-04-23

**Authors:** Rongzong Ye, Zhenhua Mai, Xiaoyan Pan, Shuting Cai, Liehua Deng

**Affiliations:** Department of Critical Care Medicine, Affiliated Hospital of Guangdong Medical University, China.

**Keywords:** acute fatty liver of pregnancy, multiple organ dysfunction syndrome, severe acute pancreatitis

## Abstract

**Rationale:**

Acute

fatty liver of pregnancy (AFLP) is a potentially fatal obstetric emergency characterized by acute hepatic failure secondary to fatty infiltration. The resultant effects include coagulopathy, electrolyte abnormalities, and multisystem organ dysfunction. Pancreatitis typically develops after the onset of renal and hepatic dysfunction. Pancreatitis has been suggested as a poor prognostic indicator because it is associated with more adverse outcomes.

**Patient concerns:**

A 29-year-old Chinese woman at 34.7 weeks pregnancy was admitted to hospital due to paroxysmal hypogastric pain and massive colporrhagia for 1 day.

**Diagnosis:**

Laboratory tests revealed hepatic and renal impairment, coagulopathy. Thoracoabdominal computed tomography (CT) scanning showed pleural and peritoneal effusion, fatty liver, and pancreatitis. She was diagnosed with AFLP, severe acute pancreatitis (SAP), multiple organ dysfunction syndrome (MODS), and intrauterine fetal death.

**Interventions:**

The patient was treated with blood component transfusions, plasma exchange combined with renal replacement therapy, antibiotic de-escalation, gastric and pancreatic secretion inhibitor, and enteral nutrition.

**Outcomes:**

After successful management, the patient was discharged without any complications on day 35 of admission. At 10 months follow-up, thoracoabdominal enhanced CT revealed was normal and laboratory tests revealed normal liver and kidney function.

**Lessons:**

Once AFLP is highly suspected or confirmed, the pregnancy should be terminated in time and active symptomatic management should be given.

## 1 Introduction

Acute fatty liver of pregnancy (AFLP) is a rare but life-threatening complication that typically occurs in the third trimester of pregnancy. Extant studies show the low incidence of AFLP ranging from 1/7000 to 1/20000.^[1]^ Maternal mortality is 10% to 15%, and fetal mortality is up to 20%.^[2,3]^ AFLP is an idiopathic disorder characterized by hepatic microvesicular fatty infiltration during pregnancy, resulting in hepatic and renal dysfunctions, coagulopathies, pancreatitis, encephalopathy, fetal acidosis, and multiple organ injuries.^[4,5]^ Its etiopathogenesis may be involved in estrogen excess, fatty acid metabolism derangements, or mitochondrial dysfunction.^[6,7]^ Pancreatitis is a potentially fatal complication of AFLP, and all patients with this diagnosis should be screened for the abnormality.^[8]^ The severity of this disease underscores the need for early diagnosis and management.

The clinical diagnosis of AFLP is challenging, and the differential diagnoses includes other peripartum conditions such as severe viral hepatitis, pre-eclampsia, hemolysis, elevated liver enzymes, and a low platelet count (HELLP) syndrome or thrombotic microangiopathies. The primary treatment for AFLP includes rapid pregnancy termination and symptomatic therapy. Liver transplantation has been considered a last resort, but its use remains controversial. We described a case of AFLP and severe acute pancreatitis (SAP) who was successfully treated after active symptomatic management.

## 2 Case presentation

A 29-year-old otherwise healthy woman at 34.7 weeks of gestation presented to a municipal hospital with paroxysmal hypogastric pain and massive colporrhagia for 1 day. Conventional prenatal examinations including blood sugar, blood pressure and urine protein were unremarkable in a local hospital. She had nausea, vomiting, and jaundice at 25-week gestation, but these symptoms spontaneously alleviated without taking any medicine. Four years ago, she gave birth to a live male baby by cesarean section.

**Figure 1:**
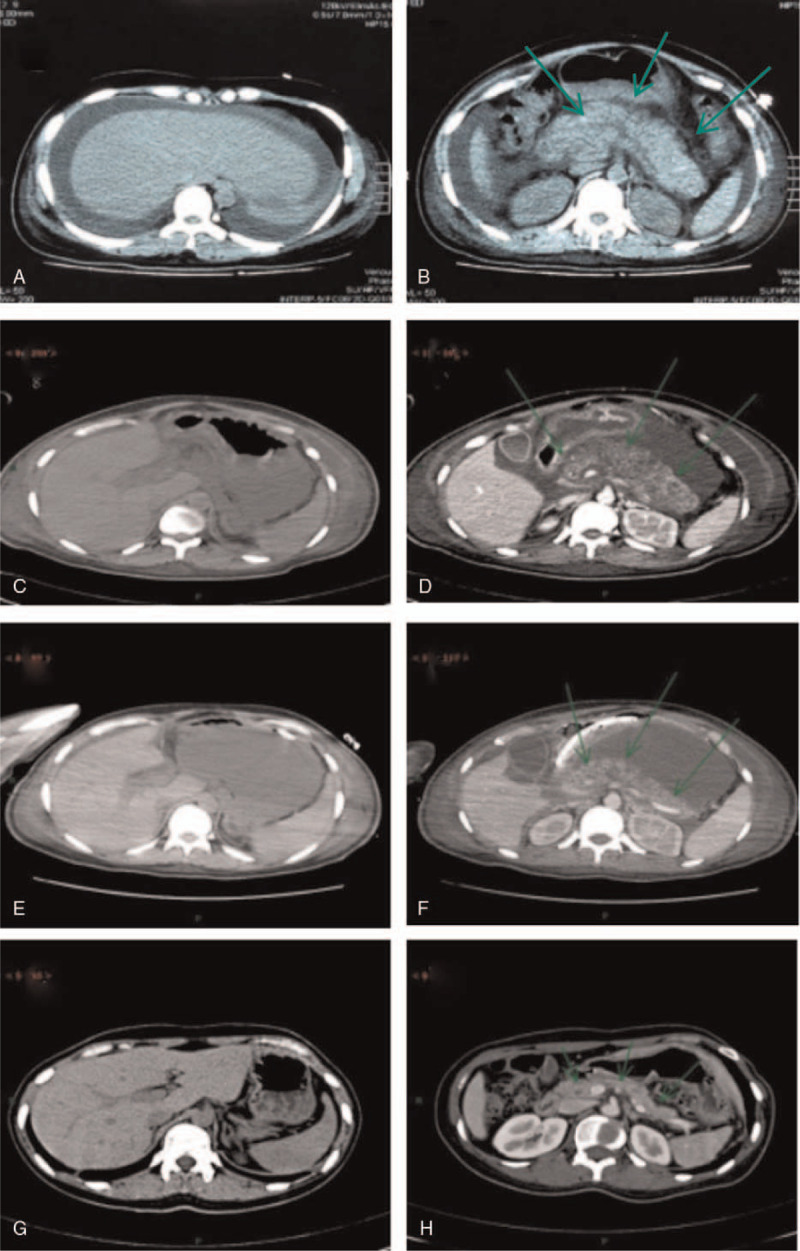
Abdominal computed tomography findings: (A) diffuse decrease of hepatic density. (B) pancreatic swelling, unclear pancreatic duct, blurred peripheral fat spaces, and peripheral foci of exudation. (C) normal size, shape and density of the liver. (D) obvious pancreatic swelling with reduced pancreatic parenchymal density and lytic necrosis. (E) homogeneous density of liver parenchyma. (F) reduced volume and uniform density of pancreas. (G) normal size, shape and density of the liver. (H) uniform pancreatic density.

On admission, blood pressure, pulse and temperature were 142/105 mmHg, 81 bpm and 36.6°C respectively. Physical examination showed gingival and vaginal bleeding, mild mucocutaneous jaundice, absence of fetal heart sound, and opening uterine. She progressed to a spontaneous vaginal delivery of a dead boy 20 min after admission, with normal placental expulsion, poor uterine contraction, and about 700 ml of postpartum haemorrhage within 2 h. Laboratory tests revealed leukocytosis, thrombopenia, hepatic and renal impairment, hyperbilirubinemia, hyperuricemia, abnormal coagulation function, positive plasma protamine paracoagulation, and normal serum amylase and lipase (Table 1). Hepatitis A, B, C and E was negative. On day 2, thoracoabdominal computed tomography (CT) showed pleural and peritoneal effusion, fatty liver, and pancreatitis (Fig. 1 A, B). The initial diagnosis included AFLP, severe acute pancreatitis (SAP), postpartum haemorrhage, multiple organ dysfunction syndrome (MODS), and intrauterine fetal death.

**Table 1.** Laboratory results in hospital stay.

	Day1	Day2	Day3	Day4	Day5	Day6	Day7	Day11	Day35	10months
WBC, × 10^9^/L	14.9	33.28	37.12	32.01	30.04	53.67	31.36	13.68	5.02	6.26
HGB, g/L	135	90.4	90.9	87.2	83.3	91.3	61	75	84.4	135
Platelets, × 10^9^/L	91	30	94	92	80	24	43	61	419.7	168
Serum AST, U/L	126.8	49.1	29.3	30.7	48.4	90.7	64.8	70.7	36.4	23.2
Serum ALT, U/L	223.3	67.1	40.7	31.2	26.6	27.5	18.5	19.4	38.3	21.3
Serum total bilirubin, μmol/L	146.1	134	112.7	139.1	147.7	195.3	110.9	147.4	11.1	12.4
SerumTG, mmol/L	1.4	0.57	1.8	2.98	3.77	4.32	1.69	2.5	1.48	0.93
Serum amylase, U/L	48	32	33	67	92	1091	1083	42		
Serum lipase, U/L	86.7	59.4	62.6	168.8	291.7	3468.2				
Serum uric acid, μmol/L	713.5	625.3	620.7	672.3	730.1	799.4	418.4	156.9	104.8	355
Serum glucose, mmol/L	4.6	9.01	8.1	8.48	10.99	6.47	13.96	6.3	4.80	5.47
Serum creatinine, μmol/L	255	218	211	247	234	262	140	79	45	67
PT, seconds	51.2	13.9	16.3	21.8	22.1	23	25.4	11.3	12.5	
APTT, seconds	68.7	25.7	29.8	30.4	32.8	42.7	62.4	36.5	34.4	
Plasma fibrinogen, g/L	0.8	1.66	1.47	1.75	2.15	1.4	2.02	2.21	2.60	
PCT, ng/ml		2.75	3.07		4.04	12.68	16.12		0.128	
Lac, mmol/L		3.3				12	10.2	4	1.1	

On day 4, laboratory tests showed elevated serum lipase and normal serum amylase (Table 1). Abdominal paracentesis drainage yielded about 800 ml of yellowish fluid. However, the patient's condition gradually worsened, with hypersomnia, decreased consciousness, and incremental serum creatinine and bilirubin.

On day 6, serum lipase and amylase levels reached the top (Table 1). The patient received high-dose noradrenaline (1.2ug/kg.min) and tracheal intubation with mechanical ventilation due to shock and respiratory failure, so she was transferred to our hospital for emergent treatment. Serum total bilirubin, amylase and lipase reached 195.3 μmol/L, 1091 U/L and 3468.2 U/L respectively (Table 1). Arterial blood gas analysis showed pH 7.34, HCO_3_^-^17.8 mmol/L, BE -7.3 mmol/L, Lac 12 mmol/L, Na^+^ 127 mmol/L, K^+^3.9 mmol/L, Ca2^+^0.83 mmol/L, Glu 8.5 mmol/L, PCO_2_ 33 mmHg, and P/F 156 mmHg. Additionally, she had acute renal failure and achieved 34 points according to the APACHE II criteria. She was started on critical care echocardiography monitoring, which helps us in identifying the cause of poor lung aeration or lung edema, and in assessing LV filling pressure and ventricular function. Owing to limited treatment options, the decision was made to utilize an intensive plasma exchange protocol. On day 7, abdominal CT demonstrated normal size, shape and density of the liver, and pancreas enlargement with uneven density (Fig. 1 C, D). The patient was treated with blood component transfusions, plasma exchange combined with renal replacement therapy, antibiotic de-escalation, gastric and pancreatic secretion inhibitor, and enteral nutrition.

On day 11, she remained hemodynamically stable, and renal and respiratory parameters almost normalized, and the consciousness and liver and coagulation function were markedly improved, and she was extubated on day12. (Table 1). On day 16, abdominal CT displayed homogeneous density of liver parenchyma, reduced volume and uniform density of pancreas (Fig. 1 E, F).

The patient was discharged on day 35 of admission and returned to our hospital 10 months later with normal laboratory tests and abdominal CT (Table 1, Fig. 1 G, H). During the hospital stay, 9700 ml of fresh frozen plasma, 2900 ml of 20% human serum albumin, 20 units of cryoprecipitate, 14 units of red cell concentrate, and 84 units of platelet concentrate were transfused.

## 3 Discussion

AFLP is an obstetric emergency as it can be fatal for both mother and baby without early identification and imperative treatment.^[2]^ AFLP was once considered to be extremely rare; however, with improved prenatal care and testing, the diagnosis can be made earlier and cases with milder conditions can be identified. The pathogenesis of AFLP is not well understood. To understand the pathophysiology of AFLP, the importance of normal changes in fatty acid metabolism during pregnancy is crucial. During normal pregnancy, the oxidation of both long and medium chain fatty acids decreases physiologically, leading to an increase in maternal fatty acid levels during pregnancy, which makes patients vulnerable to the hepatotoxic effects of fatty acids.^[9]^ AFLP is characterized by microvesicular fatty infiltration of hepatocytes, caused by genetic defects in enzymes involved in fetal fatty acid mitochondrial metabolism.^[10]^ Long-chain 3-hydroxyacyl-CoA dehydrogenase (LCHAD) is one of the most common involved enzymes. The accumulation of fatty acid metabolites in fetuses with this homozygous defect has negative effects on maternal hepatocytes.^[11]^ Defects in fatty acid metabolism during pregnancy lead to liver dysfunction and coagulation dysfunction.^[12,13]^

Diagnosis of AFLP is challenging because the initial presentation is nonspecific, similar to preeclampsia and HELLP (hemolysis, elevated serum level of enzymes, and low platelets syndrome). AFLP mainly presents as vomiting, nausea, abdominal pain and other gastrointestinal symptoms. In fact, these symptoms are the main reason for patients to seek medical treatment, which is easy to be misdiagnosed as gastroenteritis. The misdiagnosis would delay treatment and cause poor prognosis. Our patient developed nausea, vomiting, and mild jaundice at 25 weeks of gestation, however, the community doctor failed to diagnose in time. The Swansea criteria have been proposed as a clinical diagnostic tool for AFLP, but it lacks specificity. To simplify and facilitate the diagnosis of AFLP in suspected early pregnancy, Vigil-de Gracia and Montufar-Rueda^[14]^ attempted to summarize the characteristics of ’AFLP-triad’, namely the clinical symptoms (nausea/vomiting, jaundice, epigastric pain), and the laboratory results (liver function abnormalities, coagulopathy, renal dysfunction, hypoglycemia), and complications (encephalopathy, ascites, coagulopathy, renal failure).However, they only show the incidence of various indicators in AFLP cases and do not show diagnostic accuracy. Recently, Yan et al showed that the sensitivity and specificity of gastrointestinal symptoms +aminotransferase +bilirubin +bile acid +APTT/PT in the diagnosis of AFLP were 97.6% and 97.1% respectively, while more markers may be not conducive to the differential diagnosis.^[15]^

Acute pancreatitis may develop with the complexity of AFLP, and the mechanism is unclear. Fatty acid metabolites are toxic to pancreatic tissue and likely play a role in the etiology of acute fatty liver of pregnancy-associated pancreatitis.^[16]^ Pancreatitis has been suggested as a poor prognostic indicator because it is associated with more adverse outcomes.^[17]^ The serum amylase of our patients was normal in the early stage and the serum lipase was abnormal earlier than the serum amylase (Table 1). Therefore, we recommend that serum lipase and amylase be examined continuously within a few days after occurrence of hepatic dysfunction. We observed that serum lipase may be a better choice for evaluating pancreatic dysfunction than amylase. Zhou reported that Pancreatitis associated acute respiratory distress syndrome causes 60%t of deaths in SAP patients within the first week of onset.^[18]^ Early clinical manifestations of pancreatitis-associated ARDS are occult and necessitate the dynamic control of the most objective indicators of gas exchange, daily analysis of the prognostic scales, application of critical care echocardiography (CCE) to evaluate cardiopulmonary function to guide clinical treatment. CCE helps physicians in identifying the cause of poor lung aeration or lung edema, and in assessing left ventricular filling pressure and ventricular function.^[19]^ Additionally, at present, there is no consensus on the optimal timing and feeding routes of early enteral nutrition in the guidelines of various countries. Traditional concepts advocate total parenteral nutrition, but there is increasing evidence to support early enteral nutrition(<48 h), maintain intestinal permeability, reduce intestinal infections, and reduce mortality.^[20]^

Timely fetal extraction is mandatory to improve both maternal and fetal prognosis.^[21]^ According to the meta-analysis of Wang et al,^[22]^ compared with vaginal delivery/induced labour, cesarean section significantly reduced maternal mortality in in cases with AFLP, with maternal mortality reduced by 44%, and also reduced perinatal mortality. There is no consensus on the mode of delivery, but in this case cesarean section is associated with lower perinatal mortality. While prompt delivery remains the primary treatment, symptoms often persist after termination of pregnancy and patients still need intensive medical support. Acute liver failure and acute renal failure are the most important and life-threatening complications of AFLP.^[23]^ Blood purification techniques such as plasma exchange and/or renal replacement therapy can be performed in AFLP patients with organ dysfunction who cannot recover in a short time after terminating pregnancy.^[24]^ Plasma exchange has been shown to improve markers of oxidative stress and apoptosis, accelerate hepatic recovery and decrease intensive care unit stay and hospitalization, but it has not been shown to have a mortality benefit.^[25]^ Plasma exchange combined with continuous renal replacement therapy in patients with severe AFLP and multiorgan dysfunction may improve clinical symptoms and laboratory results.^[7]^ Some studies^[7,26]^ have revealed that early and timely plasma exchange combined with renal replacement therapy or continuous hemodiafiltration within 0–4 days after termination of pregnancy was often effective and safe when hepatic encephalopathy and renal failure occured in patients with AFLP. Owing to the lack of data and relatively rare diseases, it is unclear whether preventive use of plasma exchange is better or delayed plasma exchange until all medical treatments fail. However, given the lack of availability of liver transplantation and the limited medical conditions in local hospitals, we recommend early consideration of plasma exchange for patients. In addition, albumin is a multifunctional protein with antioxidant, immunomodulatory and detoxification functions.^[27]^ Our patient was treated with plasma exchange combined with renal replacement therapy immediately after transfer to our hospital, and the patient's condition improved significantly 3 days later.

Infection may develop in patients with AFLP and can include sepsis, pneumonia, urinary tract infections, Clostridium difficile, and peritonitis.^[28]^ As such, antibiotic administration should be strongly considered when infection is suspected, or the possibility for progression of illness is high. When our patients developed severe acute pancreatitis, the infection index was very high, sustained moderate fever for 2 weeks, and *Acinetobacter baumannii* was cultured in pleural effusion. After 3 weeks of antibiotic de-escalation, the patients were transferred to the general ward. Liver transplantation may be a last resort for individual patients with critically ill AFLP, especially if their liver function continues to deteriorate, or if they develop encephalopathy and elevated lactate levels, accompanied by sepsis or hypoxic/ischemic liver damage.^[29]^

Ober and Lecompte^[30]^ reported that the pathological changes in the liver were reversible; the clinical process and histopathological findings clearly indicate that there is no destructive form of hepatic insufficiency. In one study of patients followed postdelivery, all demonstrated a complete recovery of liver function with no evidence of cirrhosis or chronic hepatitis, and this highlights the need for urgent management when AFLP is diagnosed and aggressive supportive care because the acute liver failure nearly always resolves with delivery of the fetus.^[31]^ Previous studies have shown that the recovery time of liver function depends on the severity of the disease, with improvement of liver enzymes within 1-2 days, cholesterol and bilirubin within 4–6 days, and acute kidney injury within 7–10 days postpartum. However, histologic changes in the liver may last up to 5 weeks.^[13]^

Early detection coupled with advancements in critical care management have changed acute fatty liver of pregnancy from being a highly fatal complication of pregnancy to a treatable entity.^[9]^ Two reasonable recommendations are AFLP screening for pregnant women at week 34^[21]^ and longitudinal monitoring for children of mothers with LCHAD deficiency^.^^[32]^ Joueidia et al studies have shown that in AFLP, prothrombin time must be carefully monitored to anticipate major maternal complications,^[33]^ although more work is needed to determine which diagnostic parameters are most specific, reliable and prognostic.

## 4 Conclusion

Severe acute pancreatitis is a potentially fatal complication of AFLP. Early recognition and diagnosis should be made as soon as possible with laboratory tests in order to reduce severe complications and improve the prognosis. Once AFLP is confirmed or highly suspected, the pregnancy should be terminated in time and the multidisciplinary integrated support should be given. In serious cases, active symptomatic management including plasma exchange combined with continuous renal replacement therapy should be done to achieve a better prognosis.

## Acknowledgments

The authors express their gratitude to the patient and her family for their support.

## Author contributions

**Resources:** Rongzong Ye, Zhenhua Mai, Xiaoyan Pan, Shuting Cai.

**Writing – original draft:** Rongzong Ye.

**Writing – review & editing:** Liehua Deng.
